# Dental Science Then and Now

**Published:** 1896-03

**Authors:** S. B. Palmer

**Affiliations:** Syracuse, N. Y.


THE
AMERICAN JOURNAL
—OF—
DENTAL SCIENCE.

VOL. XXIX. BALTIMORE, MARCH, 1896. NO. 11.

### ARTICLE I.

### DENTAL SCIENCE THEN AND NOW.

BY DR. S. B. PALMER, SYRACUSE, N. Y.

Read at the Twenty-seventh Union Dental Convention of the Sixth, Seventh and Eighth District Dental Societies of the State of New York, held at Binghamton, Oct. 29, 30 and 31, 1895.

At no time in the history of dental science has the subject of operative dentistry claimed the attention of the profession more than it does to-day. In 1839 the first dental periodical in the world was issued, entitled *The American Journal of Dental Science.* Then little was known of true dental science. Etiology of dental caries and filling teeth have been themes for discussion until now, with flattering success and fond hopes that operative dentistry was about to be placed upon a scientific basis. That we have taught that undeveloped teeth, like those of children, which need fillings at twelve years of age, are so lacking in mineral elements as to render the dentine a conductor of thermal changes, and electric currents, that nature's process of calcification is reversed, not only to cease adding to, but in some cases to take from, the lime salts already furnished. That is to say, that vital force is an agent active in highly organized teeth to hasten decay around metal fillings (gold in particular) even more, to withdraw or absorb lime salts to the devitalization of dentine, enlargements of cavities and loss of fillings. The May issue of the *Dental Cosmos* contains an article, in which all that has been claimed for dental progress up to now is considered a mistake, and we are astonished to find, according to that, there is no recognized law to govern a young graduate in the selection and adaptation of filling materials to conditions of the teeth. Quotation: "There is no basis for the selection and adaptation of filling materials to soft teeth, hard teeth, frail teeth (in structure,) or poorly-calcified teeth." I will further quote from the conclusions given in the article, to show that there is occasion for this writing. As the writer is held responsible for the introduction of the theory, which has often been declared to have no foundation in science, he feels it due the profession to know that the electro-chemical theory of dental caries has not been discussed upon the plane of animal existence, but upon the physical or chemical plane, and that figures have been produced upon the latter plane, which, in that relation, are correct, are made to apply to all the conditions relating to the subject, ignoring the basis upon which the vital theory is founded. Thus the confusion which has attended the discussion thus far. The following is quoted: "Caries of the teeth is not dependent upon any condition of the tissue of the teeth, but on conditions of their environments." In this the vital principle is denied.

Again: "The objects to be attained in filling teeth are the perfect exclusion of the causes of caries from the tissues by sealing the cavity and securing such form as will prevent lodgment of debris about the margin of the filling, and thus prevent the further action of the causes of caries." Here, too, the vital action of the pulp through the tissue is not counted a factor. Please bear this in mind. And, again: "There is no basis for the supposition that some teeth are too soft, or too poorly calcified, to bear filling with gold, or other metal in use for that purpose, since all are found to be abundantly strong."

Once more: "With our present knowledge, the only basis for selection and adaptation of filling materials to classes of cases is the individual operator's judgment as to which he can so manipulate as to make most perfect filling, considering the circumstances, his own skill and the durability of materials."

This brings operative dentistry back to the starting point of discussion twenty years ago. Not so with practice; no amount of figuring can set practice back a single year. I regret that this crisis has been forced upon us. From the first we have done all things possible to prevent it. Reconcilation has been impossible. No quarter has been shown, and according to the writings, the electrical theory is denied recognition.

Notwithstanding all this, its scientific principles remain with an increasing practical backing.

We are discussing science, and we might as reasonably expect a collision of planets as to be told by scientists that figures proved contrary results upon definite points of investigation. Science is a revelation from the Creator to the understanding of man, through matter and natural laws. The figures which have been given, and which are supposed to annihilate the electrical theory, are based upon the *physical plane*. The electrical theory and the matter under consideration is based upon *two planes above*, in the kingdom of vital existence. There is no connection of laws and forces between the two planes, except as they have been modified in their evolution from mineral compounds up to organic tissue. Every effort has been put forth to crush out the electric theory, mostly by pointed denials, stating that it is not scientific, and that there is no ground for it to rest upon. The law and conclusive contact seems unaccountable, but not unanswerable. It must have been an oversight. I cannot believe that it was intentional to mislead. Figures which are correct upon one plane have been used to overthrow a theory which has no relation to conditions under which the figures were obtained Follow this, without discovering the misapplication, the *Dental Practitioner and Advertiser*, after quoting conclusions from the *Cosmos*, triumphantly adds:

"Now, what do our advocates of 'plastics for poorly calcified teeth' and 'gold only in teeth above the average in structure,' propose to do with these figures? They cannot be ignored, and must be met." The conclusions of their author regarding the 'adaptability, of filling materials, the proper 'electrical conditions' and tooth compatability are expressed as follows:

There must be a general abandonment of the idea that the teeth of any patient are so lacking in density as to interfere with any of the materials now in use.

"And these conclusions came from a man who has made more real scientific experiment to determine the truth than all the advocates of the 'compatability' theories have ever performed, or would perform in a hundred years, if their average for the past decade is to be maintained.

"What is to be done with these demoralizing figures? How are these confoundedly annoying demonstrations to be buried? They interfere with our personal comfort.' The paper was published in May last. Up to this writing there has been no response. Are we all paralyzed? Is there no chance for squelching this modern disturber of Israel? Must we have forced down our throats the unpalatable charge that the failure of our operations is due to our own carelessness or incompetence? If so, Heaven save us from ourselves. There is enough in the above to fill our pipes for a good long smoke, and it is hoped that the fumigation will soon begin."

The above editorial is given in spirit and language well calculated to provoke controversy rather than to preserve harmony. To answer the questions accordingly would not dignify a scientific discussion. To answer in brief:

"What do our educators of plastics propose to do with these figures? They cannot be ignored and must be met." They should not be ignored. They relate to inert matter, and in that connection, they are correct and admitted. They will not be met with a collision, as desired, but will quietly pass by upon the freight track as inert matter. "How are these confoundedly annoying demonstrations to be buried? They interfere sadly with our personal comfort." We answer, bury them with the hatchet which has caused the discomfort; and there will be no more annoyance. It says, "The paper was published in May last. Up to this writing there has been no response. Are we all paralyzed? Far from it. The trial against the electrical theory has been pushed for twenty years. It was summed up in the May *Cosmos*, and we have waited until the *Practitioner* calls for a response, in substance this: have you anything to say why sentence of banishment from operative dentistry should not be pronounced upon the electric theory?

We beg leave to prove that the case has not been tried under the laws relating to it, and will go back to the first principles of matter which relate to elements; these establish primary laws and forces and carry them forward and up through the changes until they appear in vital tissue, on the plane of animal existence, where the case will be opened for a new trial. So far as books and authority go, we will use the best, and will quote from "The Conservation of Energy," by Balfour Stewart, LL. D., F. R. S., Professor of Natural Philosophy, at the Queen's College, Manchester, Eng.: "There are four planes of material existence, which may be represented as raised one above the other. These are: (1.) The plane of elementary existence; (2.) The plane of chemical compounds, or mineral kingdom; (3.)

The plane of vegetable existence; and (4) The plane of animal existence. Their relation to each other are truly expressed by writing them one above the other, thus:

4. Animal.

3. Vegetable.

2. Mineral.

1. Elements.

"Now, it is a remarkable fact that there is a special force whose function is to raise matter from each plane, to the plane above, and to execute movements on the latter. Thus it is the function of chemical affinity alone, to raise matter from plane No 1 to No. 2, as well as to execute all the movements back and forth by action and reaction; in a word, to produce all the phenomena on No. 2. which together constitutes the science of chemistry.

It is the prerogative of the vegetable life force alone to lift matter from No. 2 to No. 3, as well as to execute all the movements od that plane, which together constitutes the science of vegetable physiology. It is the prerogative of animal life force alone to lift matter from No. 3 to No. 4, and to preside over the movements on this plane, which together constitute the science of animal physiology. But there is no force in nature capable of raising matter at once from No. 1 to No. 3, or from No. 2 to No. 4, without stopping to receive an accession of force of a different kind on the intermediate plane. Plants cannot feed upon elements, but only on chemical compounds; and animals cannot feed on minerals, but only upon vegetables, or that which has been raised above vegetables"

These facts have not been considered in the conclusions, as all are based upon the physical plane. Let us follow the laws up from one plane to another and see the changes there is in matter as we ascend. Chemical force unites elements and chemical compounds are the results, which embrace everything below organic compounds. This is the physical plane; here we are taught physics, chemistry and electrolysis, as well as other forces, cohesion, chemical affinity, etc. The principles taught by the galvanic battery and its current, and the electro-plating bath, we take up to the plane No. 3, vegetable physiology. Vital force touches chemical compounds, and vegetation springs up from the lichen to the mighty forest trees, with all intermediate productions calculated to support animal life. The galvanic battery also has been organized. One pole has been located in the foliage and the other in the rootlets of the tree or plant, and a to-and-fro current passes through the stem or trunk, while the plating fluid, this plane taken, is the sap which contains all the organized elements requisite for the growth of the vegetable.

Again, vital force comes from above and touches organized matter; animal life appears from microbes to man; the latter is given will power. Man is a cosmos. The body contains organs, conductors, currents, elements, nutrition and vital intelligence, sufficient to care for each and every part of the body, to build or take down any of its tissues. To bring up our variable laws from the vegetable plane, what was foliage reappears in the *lungs* for breathing; digesting of minerals by chemical action of the rootlets appears in the stomach. The sap appears as blood, which contains all the material needed for growth and support of the system. Each organ is endowed with intelligence and force to convey material to and from points under its supervision. The oral cavity is the receptacle in which food is ground, insalivated and prepared for the stomach. The oral cavity is the only vital laboratory in which chemists are allowed to work, and the only instance in which scientists insist upon vital tissue living upon elementary food. This is done when sensitive teeth are filled with gold without protection.

Vital currents act upon organic matter on the blood or secretions, the same as physical currents do upon metals in plating, as it is known that the reversal of a current undoes the plating and transfers it to the negative pole, which before was positive.

So thought influences these currents. Thought causes blood to fill the brain. Shame brings a flush to the face. The thought of eating a lemon causes the saliva to flow to neutralize the anticipated acid, etc. Exposure of the body to cold, the change of a collar, cutting of the hair, sitting in a draft, etc., is often the occasion of a cold. This is merely the reversal of the vital current which brings the exposed parts to an abnormal condition. When an undeveloped tooth, that is one not fully calcified, is filled while sensitive, the same reversal takes place.

In teeth there are two conditions, attended with different results. When teeth have received sufficient lime salts to render the dentine a non-electrolyte, or a non-conductor of thermal changes, this occurs; the surface of dentine in contact with the metal becomes inert matter, and serves as a protector of the vital tissue. The vital force in tissue works up to the dead line to complete calcification as it would against enamel.

When the dentine is poorly calcified, devitalization goes deeper; there not being sufficient mineral matter to protect the organic, the latter decays. Nature does not repair or resist the encroachment of decaying tissue, and quite frequently the pulp takes back lime salts from dentine beneath gold fillings which seem perfect, and which have done service for years, when the inflammation of the pulp demands removal of the filling, and it is found that only the organic portion remains as a protection. I have seen cases in which all the teeth were breaking down from this reversal of the vital current. While I have not recommended the heroic treatment, there are cases where devitalization of the pulp would correct the difficulty. This derangement of nutrition is manifest in several ways. Change of climate, long sea voyages, etc., are evidence of the same principle. Ptyalism from effects of mercury is a manifestation of systemic polarity. Nature disapproves of an element.

Let us apply this principle to the absorption of the roots of deciduous teeth, which is a vital process. By pressure directly upon the end of the root the current is reversed and the material is taken away, or from one side of the root where the pressure is in that direction. The surgeon bandages a limb or part to cause absorption. Iodine is used for the same purpose.

Thus it may be seen that a gold filling in contact with highly organized dentine strikes a chill to the tissue by a breath of cold air, or cold food, or cold drinks. In young teeth lime salts seem to be withdrawn and caries follows. I will only add that gold may be adapted to the conditions of poorly calcified teeth by insulation, by the use of chloral balsam, paraffine, etc., which exclude moisture, prevent thermal change and allow nature to proceed with calcification. This is the adaptation of materials to conditions.

Gentlemen, the doctrines which we have set forth are not of modern discovery, as may be seen by the closing sentence of a paper which I read more than a score of years ago. "What I have presented may not be new to you. To me much of this knowledge is the teaching of a 'still small voice.' communicated through the magnetic needle—an expression of a force manifest in heat, light, motion, magnetism, electricity, chemical affinity, life, and the imagination adds, Divinity." It had required years of observation and study to obtain the principles given in that early day. Of one thing I am certain, and that is that this advance doctrine could not have come from any other profession or occupation than dentistry. The oral cavity is the only laboratory where minerals and metals are daily brought in contact with vital tissue, and my excuse for the persistence in bringing this matter before the profession is, that circumstances placed me in a way to gain just this knowledge, which perhaps has never been forced upon another, and which I believe it is my duty to give the profession.

In 1847 I commenced wearing a silver plate upon which were mounted nine teeth. Having previously become somewhat acquainted with galvanism and electricity from private study, my study of oral electricity began with the wearing of the plate. I soon learned that foods of various kinds produced galvanic currents disagreeable or otherwise, but still unnatural. Sulphur in eggs, the smoke of gunpowder, which in addition to sulphur contained carbon and niter, was quite as perceptible by taste as by odor. The same in breathing through the mouth near sulphur springs. Carbon, while it is yet vegetable, as in coffee roasted, broiled meat, bread toasted, griddle cakes, etc., all communicated to the plate and gave a metallic taste; nor was this all taste, but a sensation communicated through the metal, disagreeable in silver, natural in gold, lacking always in rubber.

Another lesson was to allow the surface to become oxidized or sulphurated, which almost overcame the action. This was a help to patients, as rubber was not then in use. In time gold was substituted for the silver plate, which was a decided improvement over the latter. Gold being a negative element, the action was changed from the plate to the positive elements in contact with it. Gold being a negative element, the action was changed from the plate to the positive elements in contact with it. Gold becomes charged with electricity during contact with food, which is positive or easily decomposed. The greatest objection to gold dentures as formerly made, was the difficulty in cleansing; such plates should not be worn. The next experiment was with rubber, which was very unsatisfactory on account of heat, it being a non-conductor, which also prevents taste much more than metals. These are points to be gained by actual study, by comparison in the mouth. My observation led me to conclude that there was no difference between red and black rubber on account of non-conductivity, which is the main cause of spongy gums. Then came an aluminium swaged plate with rubber mountings, which is by far the most comfortable and natural-feeling plate I have worn. Cast aluminium had a more severe trial, as it came in contact with two bridges worn below, occluding with the aluminium caps which covered a molar on either side for the purpose of increasing the bite. The plate was inserted and I noticed a metallic taste, but thought it might pass away. On going upon the street in the evening I noticed that all the lights were remarkably unsteady, annoyingly so, but soon learned that it was caused by closing the jaws. The shock which I had not noticed before became visible or perceptible through the optic nerve. The gold, as it does in all mouths, stands at a potential higher than the positive elements in the mouth. On closing the circuit the running down of the potential caused the twitching of the nerve, and thus the apparent flickering of the lights. Removing the caps and substituting rubber corrected the trouble. Still, cast aluminium worn with gold gives more galvanic action than rolled plates. I know that it is not right to wear an upper and lower plate of different metals, except gold and platinum. Many experiments have been made that no one not in the study of the mouth would have thought of. A molar which was clasped with gold had a coronal cavity nearly in the center. This was filled several times. Being surrounded with sound enamel, there was no danger of decay. A gold filling produced no sensation when the tongue bridged from filling to clasp, or when it came between the bridge below and the filling. An amalgam filling gave a decided current. Copper amalgam was tested, and for a time was altogether the most disagreeable, but in a few months it turned black and became tasteless. This filling was all right when the tooth was extracted on account of absorption of socket. It was clasped and supported a plate forty-one years. The above are some of the lessons learned from nature direct, and to me they have become so scientifically correct that no figures upon experiments made on the chemical or second plane can set them aside. They amount to positive knowledge.

Now, let us take a stand upon the plane No. 4, where operative dentistry belongs, so far as the treatment of normal vital teeth is concerned. And here we have only kind words for "this modern disturber of Israel." As the article states, the investigations relate to the physical properties of the teeth, and are as correct as it is possible to obtain. The vital principle of the teeth seems to be ignored, as the quotation shows. "Caries of the teeth is not dependent, upon any condition of the tissues of the teeth, but upon the conditions of their environment." When teeth are extracted, that moment they become inert matter and belong to the physical or chemical plane. Crowns of pulpless teeth in the mouth, and those that have done receiving nourishment from the pulp, also come under the line of investigation so laboriously performed and scientifically set forth. All other teeth from infancy to old age are vital organs, in proportion as they receive vital support and circulation, and they belong to the fourth plane. From first to last investigators have left the vital distinction out, and by making the laws which are limited to the physical plane cover the whole ground, leaves nothing for the vital principle to stand upon. The motive for doing this I cannot discover. The greatest mystery is that it is done by the acknewledged leaders in the profession. This is the part that I most regret. After having failed to have this oversight corrected, so as to preserve harmony, a crisis has been reached, and we were compelled to make the above statement. We remained silent and the taunting question was put: "Are we all paralyzed? Is there no squelching this modern disturber of Israel?" If these conclusions gave us any practical benefits above our present attainments, it would be to an advantage; but no, as has been previously stated, the object has been to show the fallacy of the teaching which is followed by many operators.

Please note the principles we claim as beneficial to operative dentistry upon the animal plane. Trusting that the distinction has been plainly given, we will deal only with teeth in a condition in which they are nourished by vital forces through the pulp or pericementum. Since the age of twelve years has been mentioned, let us take the average teeth in that condition, which will also apply to any age, according to their vitality. The first conclusion we notice is this: "There is no basis for the supposition that some teeth are too soft or too poorly calcified to bear filling with gold or other metal in use for that purpose, since all are found to be abundantly strong." Again: "There is no basis for the selection and adaptation of filling materials to soft teeth, with our present knowledge. The only basis for the selection and adaptation of filling materials is the operator's judgment as to which he can most perfectly manipulate."

In answer to the above, I think that the majority of operators will call such teeth soft and poorly calcified. We will still use those terms upon the vital plane. It is impossible for me to harmonize the conclusion that there is no basis for the selection and adaptation of the filling materials, etc. "With our present knowledge the only basis for the selection and adaptation of filling materials is the operator's judgment as to which he can most perfectly manipulate." This is the hardest blow ever dealt to dental progress. It admits that operative dentistry is not a science, that there are no known laws by which to adopt filling materials to the conditions of the teeth. It gives license to the young graduate to gratify the highest ambition of every enterprising young operator to do gold filling whenever a tooth would seem to stand the operation, only to find to his sorrow and to the patient's loss that a mistake had been made.

Twenty-five years' experience on the Dental Examining Board of the Dental Society of the State of New York, and sixteen years of Registration for six counties in the State, has afforded knowledge of the teachings of various colleges throughout the State, as well as the understanding of graduates bearing upon this subject. With the schools, as with practice, there has been evolution. At first but a single college ventured to brave the criticism resulting from the teaching of this heretical doctrine; others have cautiously taught liberal ideas as practice led and gave support. Notwithstanding, if graduates can be relied upon, many schools are impressing their students with the falsity of the electric theory.

The most charitable allowance for opposition by investigators would be that they do not comprehend the laws and forces upon the plane of vital activity. This seems evident from the fact that the author of the article twice alludes to constitutional causes, but in a manner to act upon the environments of the teeth through saliva rather than from within, when in fact both agencies are at work at the same time. I believe that if the author had made his investigations from the chemical standpoint, together with the electro vital experiences which have been forced upon the writer, that operative dentistry would now be standing upon a scientific basis, and dental schools would be teaching laws and principles which would enable graduates to see the end from the beginning of their operations, rather than to be told to rely upon their own judgment without experience to guide them, which would be a decided gain over the claims of the author, quoted as follows:

"The greatest good that I could expect to come from this laborious investigation (except as it may effect the future study of the caries of the teeth), would be the general abandonment of the idea that the teeth of any patient are so lacking in density as to interfere with reasonable filling operations with any of the materials now in use." Thus the effect seems to have been put forth to destroy a theory without substitution. It seems time now that the coming investigators who have been watching the outcome of the electric theory, the microbic theory, the physical theory, come to the front and do some independent thinking for themselves and the profession. I am positive that great good will grow out of this occasion.

To recapitulate: Chemical force raises elements to the second plane, which is the plane of chemical compounds under the control of physical laws and forces. Vital force raises chemical compounds to the third plane, which is the vegetable kingdom. In this kingdom the matter is organic, and is under control of vital forces which act upon organic matter upon this plane. From the third plane animal force raises organized compounds up to the fourth plane, which is the plane of animal physiology. Man stands upon this plane, a cosmos. In him are incarnated all the elements and forces found in the body. All have been lifted up by a force or power from the plane above. Man has vitality, thought, will, sensibility and action. These endowments control his system, and the various organs of the body perform their functions under vital direction, involuntary or voluntary, as the occasion of circumstances demand. All the matter in the body proper, or the food which supports it, has been so organized as to be under vital control.

Here we search our book in vain for further knowledge; and here, too, investigators bring up physical forces from the chemical plane, and by them condemn vital action.

To illustrate: Many years ago the highest recognized dental authority in the world proved to the profession that a tooth was not an electrolyte, because it is a non-conductor and could not be injured by a gold filling, and that settled the matter for all subsequent investigators. Now comes up an array of figures from the same plane, and the conclusion from their teachings seem to have settled the annoying question in the mind of another defender of operative dentistry against heretical doctrines, and he exultingly remarks: "Are we all paralyzed? Is there no chance for squelching this modern disturber of Israel?" Could he have forgotten that "there is a God in

Israel?" I know in whom and in what I have trusted, and regard this as the brightest day in the history of operative dentistry.

We again call up the human body and apply our organized laws and forces to the teeth, and, to be charitable, will divide with the opponents of our theory, giving to the latter all teeth that have been extracted, pulpless teeth in the mouth, as well as those from which for any cause, either by age or abnormity, have lost vitality or pulp nourishment. This class belongs to the second, or physical plane, upon which the experiments were conducted and with which they agree in the mouth with conclusions as claimed by the figures. The other class includes the vital organs, in which the pulp and the tissue are in a normal condition.

To abbreviate: I will take for an illustration the teeth of a child, although the same principle applies to all this class of teeth, according to their organic construction. Each tooth is a vital body, a part of the whole system. It has a pulp, which is supplied with material to furnish this tissue with all that is required to make a normal tooth. It also has vital force like other members of the body, which take the material to and from the parts according as vital energy directs. We understand that an exposure of even a small portion of the body will produce a cold, perhaps neuralgia, which is a reversal of the vital current, causing an abnormal condition.

We understand that a metal filling when placed in contact with vital tissue also reverses the current. Nature will not convey lime salts to a point of contact with metal. There will be a lamina of dead matter in thickness according to the conductivity of the dead tissue. In normal dentine, and even in the more highly organized, this devitalized lining will prove a protection, and the process of calcification go on under the cover, but when the organic matter is so great as to allow fluid circulation, chemical action ensues and caries is the result. The tissue becomes an electrolyte under the laws governing the action upon the vital plane. What is understood by the adaptation of filling materials to the condition of teeth is this:

Nature will not tolerate the repeated shocks or thermal changes produced by metal in the teeth where lime salts are being deposited. Plates may be worn, or crowns upon roots, in which case there is no calcific process going on; but thermal changes produce an abnormal condition, with results as described.

When there is a liability that such changes will take place, a non conducting filling like gutta-percha, a lining of chloro-percha, or chloro-balsam, or some oxide, will allow the process of calcification to proceed. This is the adaptation which is rejected. Not only does the principle apply to the teeth, but to the whole system. Constitutional effects of mercury change the natural potential of the system, and ptyalism is the result; the whole secretions being changed. Change of climate, altitude, sea air., etc., all come under the same laws for benefits or injuries, according to conditions.

There can be no progress in etiology of dental caries so long as investigations are based upon the physical plane. The study must be conducted by observation of vital forces upon matter, the plane where man lives, moves and has his being. Then there will be harmony with cause and effect. Then we can understand how vital force controls organized matter. Also, that constitutional changes through vital circulation in the tissues is as truly a dyscrasia as gout or rheumatism.—*Dental Practitioner and Advertiser.*

---

ARTICLE II.

